# Mr. Scott on Lavements

**Published:** 1830-04-01

**Authors:** 


XXVIII.

Commentaries on the Use of Lavements. By Mr. J. Scott, Surgeon.*

A residence in France, where the glyster pipe is as common as the tea-pot in England, first led Mr. Scott to pay attention to the subject of lavements, as a simple and salutary mode of soliciting the intestines to evacuate their contents. Reflection led to observation, and this last to a small but useful volume. Mr. Scott thinks that the employment of lavements fell off greatly, and somewhat abruptly, about half a century ago—for, down to that period, they are recommended in every book, and for almost every disease. The modern disuse of this remedial agent in

---

* Octavo, pp. 186. 1829.

England, he thinks, is principally owing to the "ardour manifested in the prosecution of researches into chemical pharmacy and of therapeutical experiments." It is Mr. Scott's wish to recal the profession to a just appreciation of enemata, and to a sense of the injury which is done to the stomach and bowels by repeated and drastic purgatives. Mr. S. criticises, in a brief chapter, the various instruments for exhibiting glysters, and comes to the conclusion, that Read's syringe is the best. For our own parts, we firmly believe that no apparatus yet invented is superior to the common pewter syringe. But, as neatness, portability, and beauty will always carry the day, even in the humble fashion of a glyster-pipe, we have no doubt that Read's will be triumphant. We agree with Mr. Scott, that the rectum and colon might be made the channels for exhibiting many important medicines, with greater advantage than by the mouth. Opium has a much better and a much greater effect when thus employed than when taken into the stomach. It might be desirable to test the operation of ergot of rye in this way. After various remarks on constipation, and the different diseases to which lavements are more particularly adapted, Mr. Scott gives some sensible and useful directions respecting the kind and modes of employment. These we shall notice in a brief manner.

1. To remove costiveness merely, the object is to break the hardened and accumulated fæces by warm water, or soap and water, a table-spoonful of soft soap to the pint of fluid. Water gruel, linseed tea, or other mucilaginous fluids, may also be employed, or olive oil, treacle, sugar, &c. This is the domestic enema.

2. The temperature of the injection should be rather above that of the body, but not much. It should never be so hot as to cause pain, for then it is thrown off too soon.

3. The quantity of fluid is of some consequence. Most people err in this respect. They use too small a quantity. It should never be less than a pint—and often as much as two or three pints. The liquid should pervade the whole of the colon, or even pass the valve. It then stimulates by its temperature, fluidity, and the distension it produces, thus exciting, not only the colon and rectum, but, by sympathy, the whole line of small intestines. If the injection be slowly pushed up, we may easily introduce two or three pints of warm water, before the nisus to throw it off commences. Mr. Scott injected into the bowels of a small female, post mortem, seventeen pints of water, and even then the intestines were by no means forcibly distended.*

4. The best time for the exhibition of enemata is the morning, after breakfast. It should be retained till a pretty smart desire to expel it occurs. It is better even to let it remain and be absorbed than to throw it off ineffectually. The slower the water runs up, the more will be received. A sudden distention of the rectum and colon generally causes a sudden re-action and expulsion. The first impulse should always be resisted.

5. Mr. Scott says the bladder should be emptied before the injection is thrown up, as a full bladder sometimes obstructs the operation. In ordinary circumstances, however, this is not essential.

6. It is hardly necessary to state, that various aperient substances may be added to water, when the simple injection fails. Extract of colocynth—salts—common salt—castor oil, &c. may be used in various quantities, according to the nature of the case.

Mr. Scott has appended a copious list of formulæ for the different enemata; and, upon the whole, has produced a book which we should be glad to see as popular as possible, since its diffusion among the public at large would greatly diminish the repugnance which the people of these isles now manifest towards a very useful and important remedy.

---

* While this sheet was passing through the press, a medical gentleman of this metropolis narrowly escaped death from obstruction of the bowels, and was saved by an injection of at least six or seven pints of soap and water, which passed the valve of the colon, and gave free vent to the accumulated fæces.—Ed.

